# Development of effective human factors interventions for aviation safety management

**DOI:** 10.3389/fpubh.2023.1144921

**Published:** 2023-05-05

**Authors:** Wesley Tsz-Kin Chan, Wen-Chin Li

**Affiliations:** Safety and Accident Investigation Centre, Cranfield University, Cranfield, United Kingdom

**Keywords:** safety management system, risk assessment, Human Factors Analysis and Classification System, power distance, safety promotion, Human Factors Intervention Matrix

## Abstract

**Introduction:**

In the aviation industry, safety management has moved away from capturing frontline failures toward the management of systemic conditions through organizational safety management systems (SMS). However, subjective differences can influence the classification of active failures and their associated systemic precursors. With levels of professional experience known to influence safety attitudes, the present research examines whether experience levels among airline pilots had an impact on the classification of causal factors using the Human Factors Analysis and Classification System (HFACS). Differences in the paths of association between categories were evaluated in an open-system context.

**Method:**

Pilots working in a large, international airline were categorized into high (≥10,000 total flight hours) and low (<10,000 h) experience groups and asked to classify aircraft accident causal factors using the HFACS framework. One-way ANOVA tests were carried out to determine experience effects on the utilization of the HFACS categories, and chi-squared analyses were used to assess the strength of association between different categories within the framework.

**Results:**

Results from 144 valid responses revealed differences in the attribution of human factors conditions. The high experience group was more inclined to attribute deficiencies to high-level precursors and found fewer paths of associations between different categories. In contrast, the low experience group presented a greater number of associations and was comparatively more affected by stress and uncertainty conditions.

**Discussion:**

The results confirm that the classification of safety factors can be influenced by professional experience, with hierarchical power distance impacting the attribution of failures to higher-level organizational faults. Different paths of association between the two groups also suggest that safety interventions can be targeted through different entry points. Where multiple latent conditions are associated, the selection of safety interventions should be made with consideration of the concerns, influences, and actions across the entire system. Higher-level anthropological interventions can change the interactive interfaces affecting concerns, influences, and actions across all levels, whereas frontline-level functional interventions are more efficient for failures linked to many precursor categories.

## 1. Introduction

Safety management in aviation integrates the concepts of system safety with human factors and human performance in system design and operation. In airline operators, the safety management function is responsible for the integration of safety-related activities across different parts of the organization through a safety management system (SMS) framework. Industry best practice follows a generic framework with four major elements—*Safety Policy and Objectives, Safety Risk Management, Safety Assurance,* and *Safety Promotion*—all of which are generally held within organizational boundaries (1).

Aviation safety management has moved away from focusing on frontline errors and toward focusing on systemic threats held dormant elsewhere in an organizational system (2). However, subjective biases can affect which systemic threats are selected as inputs to the SMS framework. To illustrate, the first SMS element of *Safety Policy and Objectives* establishes standards and targets in relation to threats and precursors. These factors in turn informs the elements of *Safety Risk Management* and *Safety Assurance*, such as by plotting the likelihood of occurrence of each factor against the severity of the potential consequences in a safety risk matrix to calculate and manage the level of safety risk (1). Although human error frameworks have been devised to assist in the classification and evaluation of threats and precursors, the selection of factors for inclusion is still dependent on the context or environment within which they are interpreted, and the appraised risk levels may be influenced by what the investigator subjectively considers to be relevant and tolerable (3). Unless an understanding of the values and beliefs guiding the assessment of threats and precursors are obtained, the issue is that it will remain difficult to ensure that SMS frameworks are adequately encompassing.

As experience within a professional environment is known to influence the values, pattern identification, and problem assessment strategies among individuals (4–6), the first goal of the present research is to investigate how experience levels among analysts can influence the classification of causal factors. In addition, as the initial analysis of causal factors will, in most cases, be used to inform the creation of intervention strategies in the final *Safety Promotion* element of the SMS framework (7), an understanding of how different people view interactions between threats and precursors will help to ensure that interventions to rectify causal factors are considered to be relevant by the target audience. Thus, the second objective of the present research is to determine whether high and low experience groups differed in their identification of paths of associations between active failures and latent conditions. The purpose of this research is to inform improvements in safety management by gaining an understanding of how professional experience can affect the classification and evaluation of errors, threats, and their associated latent conditions.

## 2. Literature review

### 2.1. Aims and objectives

The present research attempts to bridge the gap in the interface between two concepts. The first concept is that acculturation to a professional environment will alter individuals’ classification of threats and precursors into categories. The second concept is that the paths of association between these categories are reflective of individuals’ beliefs of relevance between active failures and latent conditions in the cause-and-effect relationship of human factors faults. The identification of threats and precursors functions as the input factor for safety management. An understanding of how professional influences can affect how these factors are classified and associated with each other will be useful for safety managers in the development of relevant human factors interventions for SMS implementation.

The Human Factors Analysis and Classification (HFACS) framework provides a taxonomic approach for the classification of active failures and latent conditions in the lead up to an incident or accident (8). The structured, taxonomic nature of HFACS enables the comparison of classification consistency between groups of analysts, as well as the examination of associations between the categories and levels within the taxonomy. As this paper was part of a project evaluating SMS in the flight operations department of an airline, acculturation to the professional environment was measured by comparing the respondents’ flying experience in total flight hours. In cases where two or more categories were associated to a significant degree, their interactive contexts were reviewed in an interactive open-system model (9). Practical differences in the development of safety interventions were also reviewed in the context of organizational practices and attitudes to assist in the selection of more viable intervention strategies.

Thus, the research questions are:

1. Does professional experience (as measured in total flight hours) influence the classification of safety factors into HFACS categories?
2. Do the paths of association between HFACS categories differ between high and low experience groups?
3. In cases where significant associations between two categories are identified, how can SMS practitioners select more favorable human factors interventions?

### 2.2. The HFACS framework and human factors interventions

The Human Factors Analysis and Classification System (HFACS) was developed for analyzing human error based on the interrelationships between active failures and latent conditions (Table 1). Causal factors are classified into a structured framework including 18 categories across four consequential levels (L1–L4). Starting from classifying causal factors into active failure categories at the lowest level, analysts work upwards through the framework to classify the associated precursor latent conditions using the presented taxonomies (8). After the active failures and latent conditions related to an occurrence have been classified into HFACS categories, the inter-relationships between the categories can be statistically identified. Mitigation strategies can then be created for each condition by five different types of intervention approaches as proposed in the Human Factors Intervention Matrix (HFIX) (10). These include organizational/administrative; human/crew; technology/engineering; task/mission; and operational/physical environment approaches. Evaluation criteria of cost, acceptability, feasibility, effectiveness, and sustainability can then be applied to each intervention to assess the likelihood of success (Table 2). By pitting HFACS failure categories against the five types of intervention approaches in HFIX, the comprehensiveness of SMS intervention strategies can be strengthened by ensuring that a wide array of possible interventions is considered. The idea is to comprehensively utilize as many categories as possible and satisfying as many evaluative criteria as possible, to ensure the breadth and depth of human factors interventions (10).

**Table 1:** The Human Factors Analysis and Classification System (HFACS) framework.

Level 4: organizational influences	Resource management organizational climate organizational process
Level 3: unsafe supervision	Inadequate supervision Planned inappropriate operations Failed to correct a known problem Supervisory violations
Level 2: preconditions for unsafe acts	Adverse mental states Adverse physiological states Physical/Mental limitations Crew resource management Personal readiness Physical environment Technological environment
Level 1: unsafe acts of operators	Decision errors Skill based errors Perceptual errors Violations

**Table 2:** Intervention approaches and evaluative criteria proposed in the Human Factors Intervention Matrix.

Intervention approaches	Evaluative criteria
Organizational/Administrative Human/Crew Technology/Engineering Task/Mission Operational/Physical environment	Cost Acceptability Feasibility Effectiveness Sustainability

Deficiencies in the HFACS framework can make the subsequent development of intervention strategies problematic. One problem arises from the fact that all four levels of HFACS are contained within the boundaries of a closed, organizational system. If the outcomes of the closed-system analysis are used exclusively as the basis for developing human factors interventions, then the resulting interventions will become narrowly focused on organizational solutions. High level organizational solutions (i.e., HFACS L3–L4) were found to have limited control over the frontline work processes (11). Organizational processes can be affected by a range of occupational factors external to the organization, and employees within organizations can simultaneously belong to many cultural groups (12). It was therefore considered necessary to investigate whether the demographics of HFACS analysts had an influence on how causal factors are classified within the taxonomy.

The second problem is that the application of HFACS data requires the statistical examination of paths of association between categories. These paths of association are reflective of how analysts assessed the inter-relationships between active failures and latent conditions. Interventions to rectify a specific failure are likely to be evaluated as more favorable if they are directed at associated latent conditions. With multiple paths of association, it may be possible for an active failure to be resolved through a range of intervention methods spread across several categories. While previous research has identified direct associations between latent conditions at the higher organizational and supervisory levels (L3 and L4) with active failures at the frontline (L1) (13), these paths of association may differ among analyst groups. Socio-economic contexts, for example, were considered to directly affect the actions of frontline workers at HFACS level 1 without influencing the higher-level systemic conditions (11, 14). Therefore, in combination with the likelihood of demographic effects on the classification of causal factors within HFACS, it was also imperative to determine whether the paths of association between categories differed between groups.

### 2.3. An open-system model of safety and professional values

Although the identification of paths of association using HFACS enables SMS practitioners to direct human factors interventions toward associated categories, it is not always possible for organizations to implement every possible intervention (10). This can occur in situations where there are multiple paths of association originating from one category, or when not all precursors can be rectified due to factors such as cost or time constraints. An evaluation of the interactions between latent conditions and intervention strategies in the open-system context can assist in the selection of more favorable human factors interventions for implementation.

**Figure 1:**
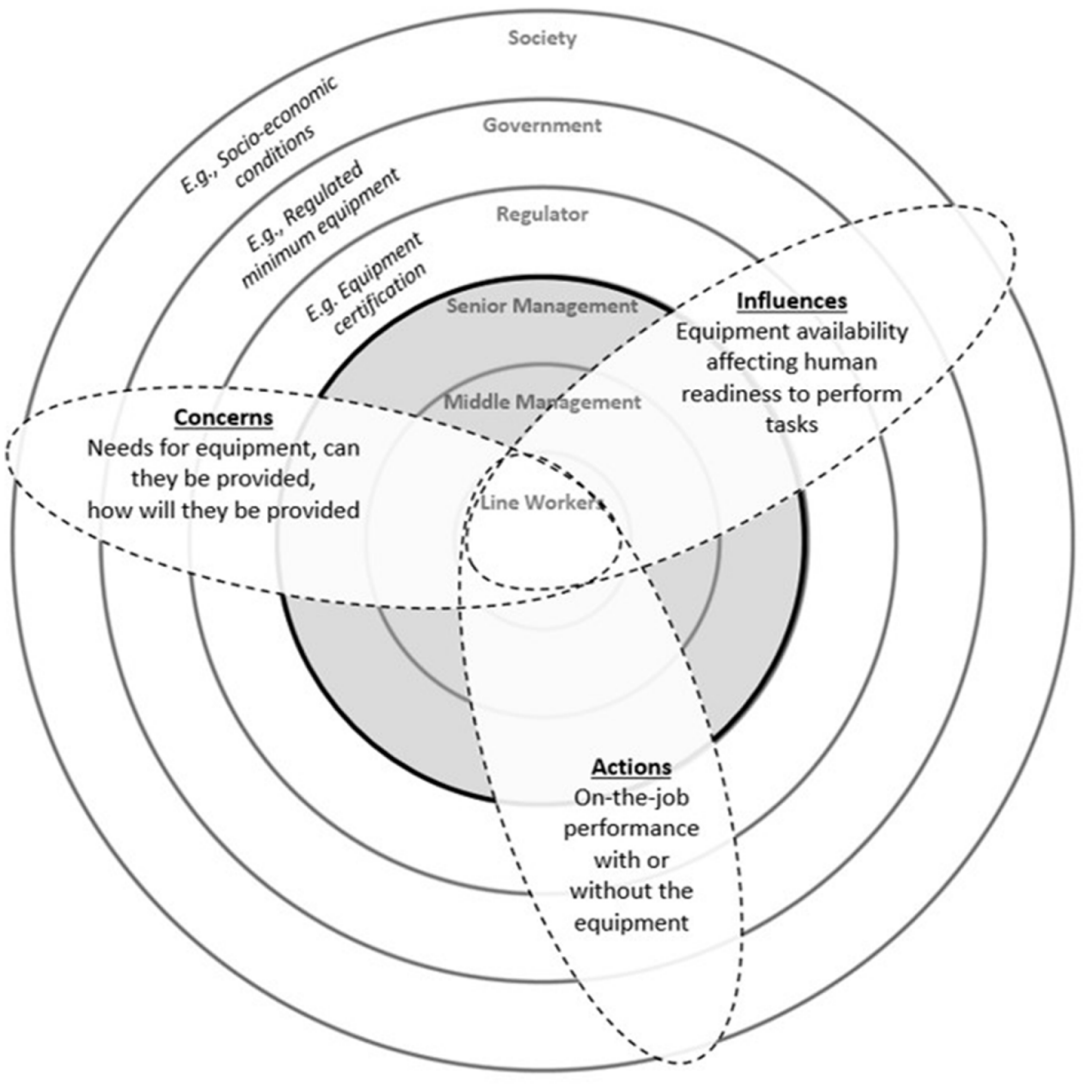
The Ripple Model of Safety Culture showing the levels of influence and meta-categories. The four levels of HFACS are contained within the central layers (shaded gray), and the meta-categories of concerns, influences, and actions act across the layers to interactively affect performance.

Based on a review of human factors influences in a range of high reliability organizations, Morley and Harris (9) presented the Open-System (Ripple) Model which extends the composition of safety culture across six layers in an open system (Figure 1). The core and the two layers immediately surrounding it are representative of organizational factors, roughly overlapping with HFACS. The three outer layers extend beyond the organization. Unlike in HFACS where latent conditions are classified into explicit categories, in the Ripple Model latent conditions transpire across the various layers through the interactive meta-categories of concerns, influences, and actions. By integrating latent conditions with open-system meta-categories, the *Safety Promotion* element of the SMS can be made more relevant to the target audience (1). For example, interventions directly focusing on active failures at the frontline are unlikely to be effective, as the *actions* of workers are still constrained by outer-layer *concerns* (e.g., regulations from society and government) and *influences* (e.g., equipment availability from management influences). In this case, if paths of association between the active failure and latent conditions have been identified, then a more viable option may be to intervene through higher-level conditions. Interventions at the outer “society” layer, for example, will concurrently cover the HFIX approaches of human/crew (actions of line workers), organizational/administrative (changes in equipment provision), and task/mission (reflective of societal expectations).

Notably, at the outermost level of the Ripple Model is the *society* layer which represents culturally-influenced values such as perceptions of risk and the willingness to pay for safety (9). In the aviation context, social influences work in association with organizational influences on influencing risk-taking behaviors (15). Although these social values are typically considered as manifestations of national culture, professional training and experience within occupational environments are also known to indoctrinate cultural responses. In aviation, these occupational factors include shared training, social and physical distance to the organization, confined work environments, occupational lifestyles, and loyalty to work interfaces (5). People with more experience within specific professions are better able to piece together information based on the identification of familiar patterns (6), and experts tend to develop strategies to cope with stress and uncertainty in problem assessment (4). It is therefore plausible that interactions between latent conditions and the meta-categories of concerns, influences, and actions will differ depending on the levels of experience and expertise. To understand how experience and expertise can subsequently affect the creation of diversified human factors interventions within the SMS, a measure to quantify operator experience needs to be established.

### 2.4. Professional experience, expertise, and organizational hierarchies

As this paper was part of a project evaluating SMS in airline flight operations departments (i.e., the pilot department), a literature review was carried out to determine how previous researchers measured experience and expertise among pilots. Possession of more advanced licenses and working for larger airlines were known to be associated with a lower incidence of error (16, 17). However, these variables are of limited relevance for organizational SMS applications where all the participants will be working in the same organization, with identical prerequisite licenses.

Individual flying experience as measured by total flight hours (TFH) has been widely employed as a measure of expertise, with higher TFH linked with declining error rates (18) and having a protective effect against violations (19). TFH was also known to affect pilots’ perception of own performance and decision-making processes, with higher flight hours related to higher scores in recognizing problems and implementing solutions (20). In addition, TFH has been championed as a more relevant proxy measure of expertise than other quantitative measures such as error rates and performance parameters. Precise aircraft control may not actually be reflective of expertise as airline flying requires a mix of cognitive, manipulative, and interactive skills (21). Experts may be doing things in a qualitatively different manner, such as by making minor sacrifices to flight control accuracy to leave cognitive space for other tasks.

TFH is also a better measure of experience at the multi-crew or system-level as it enables the comparison of power distance both between and within ranks. Between ranks, one’s organizational position is a prominent predictor of speaking-up, and seniority can reduce status barriers to challenge those in power (22, 23). It is probable that higher ranking pilots (e.g., senior Captains) will be more likely to classify latent conditions to higher HFACS levels. However, relative power distance within ranks may also affect teamwork behaviors. Todd & Thomas (24) found flight crews consisting of a high-hour Captain and a low-hour First Officer (the high power distance scenario) to have scored significantly lower on monitoring and cross-check markers than with higher-hour First Officers (same role and rank pairing, but comparatively lower power distance). Crews lower in power distance had a greater number of positive teamwork behaviors (25). The use of TFH as a measure of experience can therefore help to capture these power distance factors influencing interactive interrelations in the wider system.

### 2.5. External factors driving safety management interventions through safety culture

Based on the different groups’ classification of causal factors using HFACS, the HFIX framework (Table 2) can be applied in the development of human factors interventions that are congruent with the unique characteristics of each demographic group. However, HFIX interventions and their evaluative criteria may not be directly applicable in the organizational context. As the SMS is generally held within organizational boundaries, each organization should implement a rationalization process to evaluate intervention strategies in accordance with their own organizational context (26).

As safety culture relates to an organization’s attitudes toward safety, it provides a good starting point for assessing whether safety practices or human factors interventions are suitable. Safety culture is defined by the values, beliefs, problem-solving methods, and working practices within an organization (27). The first two criteria (values and beliefs) can be viewed from an anthropological perspective, with culture representing shared, tacit patterns of meaning that people draw on as they decide on how to behave (28). On the other hand, the last two criteria (methods and practices) can be understood from a functionalist perspective, representing explicit patterns of behaviors of the people performing the task (28, 29). Thus, safety culture is both the driver and the product of safety practices (30).

When coming up with human factors interventions, safety managers need to understand which aspect of the safety culture they are trying to change. To change the anthropological aspects, overarching changes in culture and system interactions are required to generate new values and beliefs that dictate how people behave. These will typically originate from the outer levels of the Ripple Model and act through the meta-categories of concerns, influences, and actions. On the other hand, the functionalist methods and practices can be rectified by tangible, function-based changes closer to the central line-worker levels (31). However, these function-based changes are less desirable as the possible range of improvements are ringfenced (32). To illustrate, consider the example of functional changes to the technological user interface (which would be classified at HFACS level 2). These changes will have limited impact on safety outcomes unless the supervisory (L3) and organizational (L4) levels commit to providing resources to implement relevant training and monitoring programs. Both the interactive interfaces (for anthropologic changes) and the boundary at which the ringfencing occurs (for functionalist changes) are dependent on the how the human factors interventions are implemented across levels. This will in turn affect which of the HFIX intervention approaches are covered, and how these approaches are evaluated within HFIX.

## 3. Methods

### 3.1. Participants

Participants were recruited by convenience sampling through pilot union membership lists. Ethics approval was provided by the research institute Ethics Committee (CURES 12290/2020), and data collection took place from November 2020 to January 2021. One-hundred and forty-seven (*N* = 147) airline pilots participated in the present research, including Captains (*n* = 65), Co-Pilots (*n* = 77), and Other/Not Reported (*n* = 5). Age ranged from 25 to 65 years, mean = 42.3, *SD* = 9.5. Mean flying experience was 10,268 h, *SD* = 6,661. Participants with less than 10,000 h were categorized into the low experience group, whereas their colleagues with 10,000 h or more were assigned into the high experience group. Ten-thousand hours of total flight time was selected as the cut-off point as it was the median point of the collected data, and because it coincidentally corresponded with previous studies which found the protective benefit of increased pilot experience on frontline violations to diminish after 10,000 flight hours (19).

### 3.2. Coding framework

The Human Factors Analysis and Classification System (HFACS) was employed as the coding framework for the present study. Developed from an analysis of accident reports, HFACS provides analysts with a four level framework of latent and active failure taxonomies (8) (Table 1). At the first level are categories relating to *Unsafe Acts of* Operators, representing active failures at the frontline which directly led to the accident or incident. The latent or dormant conditions which were associated with these active failures in the causal sequence can then be classified across the three upper levels covering the *Preconditions for Unsafe Acts* (L2), *Unsafe Supervision* (L3), to *Organizational Influences* (L4). A total of 18 causal categories distributed across the four levels were provided to classify specific active or latent failures. Each higher level affects the next downward level, and in theory if one of the conditions at any level is corrected, then the sequence of latent conditions is cut off and the active failure or adverse outcome will be prevented (8).

### 3.3. Research design

A survey hosted on the Qualtrics platform^1^ was used for data collection. Members of an airline pilot union were sent an anonymous hyperlink to the survey via email. The union represents the pilots working for a large international airline based in an East Asian city-state, with 70% of its members identifying as expatriates. A hyperlink in the email directed the participants to an information page describing the research purpose, ethics, data handling protocols, and participant rights. At the end of this page, the participants’ consent was sought, and if they provided consent then the webpage redirected the participants to the main survey. The first part of the survey contained demographic questions which enabled the categorization of participants according to their experience levels. The second part of the survey included the HFACS coding exercise which was based on an analysis of the official accident report of a mid-air collision between a Tupolev 154 and Boeing 757 aircraft over Uberlingen, Germany on the 1st of July 2002 (33). The accident occurring over Uberlingen was selected as the case study subject for several reasons. Firstly, it was frequently used in numerous studies on HFACS and other accident and incident investigation methodologies (34, 35). Secondly, the accident involved a plethora of system players as it occurred at the interface between different states, organizations, operators, and professions. Subsequent to the publication the official accident report, the accident has been reanalyzed from different perspectives, including technical and regulatory faults (36), situational awareness (37), and human factors and system errors (38). Participants were provided with a short description of the accident and contributing factors were presented as survey statements. Selection boxes containing the 18 HFACS categories were placed alongside each contributing factor statement. Participants were instructed to select the boxes corresponding to the HFACS category (or categories) that they considered to be the most suitable, or to select none, if they did not think that the contributing factor fit into any of the 18 HFACS categories. Each category was counted only once per response, simply as an indicator of presence, to avoid over-representation.

## 4. Results

### 4.1. Sample characteristics

After screening for missing and invalid data, 144 of the 147 total responses were included in the final analysis. In the present results, the low experience group (*n* = 67) was made up of two Captains (3.0%), 62 Co-Pilots (92.5%), and 3 Other/Not Reported (4.5%), whereas the high experience group (*n* = 77) consisted of 62 Captains (80.5%) and 15 Co-Pilots (19.5%). Hours of flight experience was therefore approximately representative of rank. The frequency of participants choosing a particular HFACS category as contributory to the Uberlingen mid-air collision is presented in Table 3. As each participant was able to select any number of HFACS categories over the course of the coding exercise, there were different levels of frequency overlap which makes the overall frequency of classification unrelated to the number of participants within each group.

**Table 3:** Frequency and percentage of participants from the low and high experience groups who indicated an HFACS category as a contributory factor in the Uberlingen accident.

	HFACS categories	Low experience (*n* = 67)		High experience (*n* = 77)	
	Frequency of classification=	574	100.0%	682	100.0%
Level 1	Decision errors	34	5.9%	27	4.0%
	Skill based errors	40	7.0%	57	8.4%
	Perceptual errors	31	5.4%	41	6.0%
	Violations	23	4.0%	26	3.8%
	Level 1 total	128	22.3%	151	22.1%
Level 2	Adverse mental states	21	3.7%	16	2.3%
	Adverse physiological states	5	0.9%	10	1.5%
	Physical/Mental limitations	53	9.2%	46	6.7%
	Crew resource management	32	5.6%	46	6.7%
	Personal readiness	17	3.0%	20	2.9%
	Physical environment	13	2.3%	7	1.0%
	Technological environment	55	9.6%	67	9.8%
	Level 2 total	196	34.1%	212	31.1%
Level 3	Inadequate supervision	17	3.0%	18	2.6%
	Planned inappropriate operations	51	8.9%	57	8.4%
	Failed to correct a known problem	32	5.6%	48	7.0%
	Supervisory violations	15	2.6%	19	2.8%
	Level 3 total	115	20.0%	142	20.8%
Level 4	Resource management	30	5.2%	47	6.9%
	Organizational climate	48	8.4%	66	9.7%
	Organizational process	57	9.9%	64	9.4%
	Level 4 total	135	23.5%	177	26.0%

### 4.2. Experience effects on HFACS classification

Based on the differences in HFACS classification between the high and low experience groups, statistical tests were carried out to determine if there was a significant effect of experience on HFACS classification across 18 categories, and also across the four levels. As the high and low experience groups were independent, and the sample sizes were approximately balanced and reasonably large (*n* ≥ 25), the assumptions for ANOVA were met. One-way ANOVA tests were performed using Minitab (version 21). The results showed that there was a significant, large effect of experience on classifying factors to the “Physical/Mental Limitations” (L2) category [*F*(1, 142) = 6.45, *p* = 0.012, *η*^2^ = 0.43]; and a significant, medium effect of experience on the “Organizational Climate” (L4) category [*F*(1, 142) = 4.37, *p* = 0.038, *η*^2^ = 0.10]. Comparatively, the low experience group more frequently classified “Physical/Mental Limitations” (L2) conditions, whereas the high experience group made more classifications to the “Organizational Climate” (L4) category (Table 3). Differences between the high and low experience groups were also found across the four HFACS levels, with ANOVA tests finding a small effect on the frequency of utilization of HFACS level 4 between the two groups [*F*(1, 142) = 4.50, *p* = 0.036, *η*^2^ = 0.05].

### 4.3. Paths of association differed by experience levels

To determine if the high and low experience groups produced different paths of association between HFACS categories, chi-squared analyses were carried out to predict the statistical strength of association between the 18 HFACS categories. High and low experience groups were entered as the exploratory (independent) variable and the presence or absence of each of the 18 HFACS categories were evaluated as the response (dependent) variable. In cross-tabulations where the chi-squared analyses were significant between two categories, further analysis using Goodman and Kruskal’s tau were used to calculate the levels of association, and odds ratios were calculated to provide an estimate of the likelihood of the participants’ utilization of one category within HFACS predicting the utilization of another category. If the degree of association between HFACS categories were dependent on the experience (TFH) of the person making the classification, then the tau statistic will assist in predicting concomitant presence across categories, and the odds ratio will present the likelihood of utilization.

**Figure 2:**
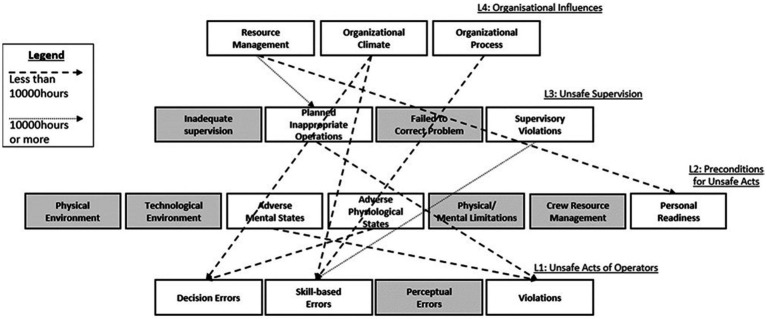
Paths between categories in the HFACS framework with significant associations (*p* < 0.05) by Chi-square analysis for low experience and high experience groups. Categories not associated with any other category are shaded in gray.

Analysis of the strength of association between the 18 HFACS categories showed different paths of association between the high and low experience groups (Figure 2). For the low experience group, seven pairs of HFACS categories had significant chi-square associations at *p* < 0.05 (Table 4; Figure 2). Categories at HFACS level 4 (*Organizational Influences*) were associated with four other categories. “Resource Management” was associated with “Personal Readiness” at level 2; and, notably, three of the four level 4 categories were directly associated with frontline “Decision” and “Skill-Based Errors” at the *Unsafe Acts* (L1) level. From level 3 (*Unsafe Supervision*), only the category “Planned Inappropriate Operations” was found to be statistically significantly associated with “Violations.” From level 2, “Adverse Mental States” and “Adverse Physiological States” were, respectively, associated with “Violations” and “Decision Errors” at level 1. For the low experience group, 8 of the 18 categories had no statistically significant associations with other categories within the HFACS framework. As presented in Table 5, very high odds ratio was discovered between “Adverse Mental States” (L2) and “Violations” (L1). The existence of one category was 4.24 times more likely when the other is present. Similarly, “Resource Management” (L4) was associated with “Personal Readiness” (L2) with a very high odds ratio of 4.27.

**Table 4:** Significant Chi-square tests of association and associated Goodman-Kruskal tau and odds ratio values between categories in the HFACS framework as utilized by pilots with less than 10,000 h of total flight experience.

Significant associations between upper level and lower-level categories in the HFACS framework (<10,000 h TFH)	Chi-square		Goodman-Kruskal tau	Odds ratio
	Value	*p*		
**HFACS level 4 with lower-level categories**				
Resource management × Personal readiness (L2)	6.138	0.013	0.0916	4.2667
Organizational climate × Decision errors (L1)	11.882	0.001	0.177	1.0357
Organizational climate × Skill-based errors (L1)	4.083	0.043	0.0609	0.2899
Organizational process × Skill-based errors (L1)	4.485	0.034	0.0669	0.1325
**HFACS level 3 with lower-level categories**				
Planned inappropriate operations × Violations (L1)	4.481	0.034	0.0669	0.2943
**HFACS level 2 with lower-level categories**				
Adverse mental states × Violations (L1)	7.062	0.008	0.1054	4.2424
Adverse physiological states × Decision errors (L1)	5.567	0.0245^*^	0.083	n.s.

^*^Fisher’s exact test.

**Table 5:** Significant Chi-square tests of association and associated Goodman-Kruskal tau and odds ratio values between categories in the HFACS framework as utilized by pilots with 10,000 h of total flight experience or more.

Significant associations between upper level and lower-level categories in the HFACS framework (≥10,000 h TFH)	Chi-square		Goodman-Kruskal tau	Odds ratio
	Value	*p*		
**HFACS level 4 with lower-level categories**				
Resource management × Planned inappropriate operations (L3)	4.084	0.043	0.053	0.2981
**HFACS level 3 with lower-level categories**				
Supervisory violations × Skill-based errors (L1)	6.005	0.032^*^	0.078	0.26
**HFACS level 2 with lower-level categories**				
Nil		n.s.		

^*^Fisher’s exact test.

For the high experience group, the results found far fewer associations across the HFACS categories (Table 5; Figure 2). “Resource Management” (L4) was associated with “Planned Inappropriate Operations” at the adjacent level (L3), and this was also the only association between categories at adjacent levels for the high experience group. “Supervisory Violations” at level 3 was associated with “Skill-Based Errors” at level 1. Across the entire HFACS framework, 14 of the 18 categories were not found to be significantly associated with any other category.

## 5. Discussion

### 5.1. Experience effects on classification hierarchy

**Figure 3:**
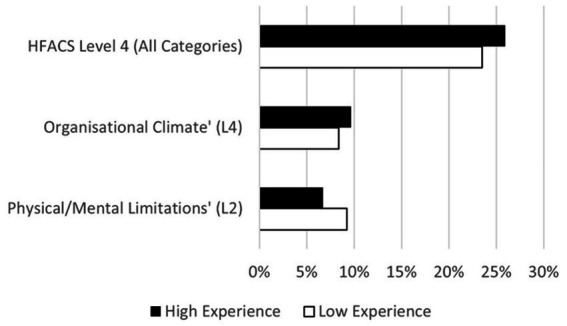
Frequency of utilization of HFACS categories and levels between high and low experience groups.

The current results showed that the high experience group was more inclined to categorize latent factors into higher organizational levels (HFACS L4), and within level 4 this was replicated in the “Organizational Climate” category (Table 3; Figure 3). This finding confirms the effects of experience on hierarchical relations and power distance. With higher experience levels considered as an approximation of lower relative power distance to the senior organizational or managerial levels (24), and that low power distance investigators were more likely to attribute failures to organizational faults (35), it was unsurprising that the higher experience group was more inclined (or less restrained) to classify human factors conditions to the organizational level (L4). Similarly, the finding of greater utilization of the “Physical/Mental Limitations” (L2) category by the low experience group corresponded with previous studies of HFACS which discovered that high power distance investigators were more likely to use lower level categories (35). The findings therefore provide support for the use of TFH as a proxy measure of experience.

The results also confirm that SMS implementation can be influenced by expertise and the ability to handle stress. Stress and uncertainty factors are represented by the conditions of operators at level 2 of HFACS (8). In the low experience group, an association with very high odds ratio was found between the latent condition of “Adverse Mental States” (L2) and the active failure of “Violations” (L1) (Table 4). This was not replicated in the high experience group. As the ability to cope with stress and uncertainty is related to expertise (4), the finding of strong association between adverse mental conditions and active failures may be explained by the low experience group’s comparative lower levels of expertise and hence their lower capacity to handle stress and uncertainty.

### 5.2. Paths of influence affecting SMS elements

In HFACS, multiple paths of association can stem from each active failure or latent condition toward other categories (13). The higher experience group in the present study had a more targeted approach in finding these paths of association than the low experience group. In the high experience group, only two pairs of HFACS categories were associated (Table 5). In contrast, the low experience group had seven pairs of associated HFACS categories (Table 4).

The two groups also classified the identical list of factors into different HFACS categories (Figure 2). At level 1, representing active failure categories, the high experience group summarily classified the causal factors into the “Skill-Based Errors” category, and these skill-based errors were solely associated with “Supervisory Violations” at level 3. In contrast, the low experience group spread the same factors across three categories of “Decision Errors,” “Skill-Based Errors,” and “Violations,” which were in turn considered to have stemmed from different latent conditions across levels 2–4. Overall, the low experience group had paths of influence across six different latent condition categories throughout level 2 (2 categories), level 3 (1 category), and level 4 (3 categories) (Table 4; Figure 2). The results therefore revealed that the high experience group was more targeted in classifying active failures and the associated latent conditions. This finding provides support for the previous knowledge that people with more experience perform better at identifying patterns of association (6). Similarly, the finding of a more dispersed classification style in the low experience group also corresponded with the previous contention that steeper hierarchies and higher power distance can lead to the fragmentation of safety culture across organizational levels (30).

Also notable was that across both low and high experience groups, only four of the nine associated pairs were at immediately adjacent HFACS levels, and the other five associations crossed more than one HFACS level (Figure 2). As the HFACS framework is conventionally applied on a level-by-level basis (8), this finding of cross-level associations can offer an improvement to how HFACS is applied in SMS applications.

### 5.3. Selecting entry points for human factors interventions

In the present research, the odds ratio between two categories provides a bilateral estimate of the likelihood of the participants’ use of one category predicting the use of another category. This statistic can assist SMS functions to identify human factors conditions which are likely to be jointly influenced by two or more conditions. As it was previously established that interventions involving high-level, outside-in mitigations may be more desirable than task or function-based changes by ensuring that the mitigations will not be limited by wider commitment, capacity, and resources constraints (31, 32), the current findings provide useful information for SMS functions to select more effective human factors interventions.

When two or more categories are linked with a high odds ratio, it may be more desirable to intervene through the higher-level category. For example, in the present results, a high odds ratio was discovered between the categories of “Resource Management” (L4) and “Personal Readiness” (L2) in the low experience group. Using the Ripple Model meta-categories to interpret the interactions with “Resource Management” as the starting point, it is possible to deduce that management-level decisions on the allocation of human, financial, equipment, and facility resources *influenced* personal readiness (8). In civilian airlines it is easy to envision that many of these resources may be controlled by factors external to the organization. In this instance, a single outside-in intervention, such as through regulatory changes to equipment provision, can concurrently cover the five HFIX approaches by altering the concerns, influences, and actions across the various levels (Figure 1). In contrast, if interventions were to be developed with “Personal Readiness” as the starting point, changes to personal behaviors will be ineffective as *actions* at the individual-level will still be constrained by the resources and influences provided by the organization. Thus, the argument is that human factors interventions starting from the higher-level category of “Resource Management” are more likely to be successful.

### 5.4. Consequences for human factors interventions in SMS implementation

In the present results, 7 of the 18 HFACS categories were not associated with any other category in either low or high experience groups (Figure 2). While it would be far-fetched to assume that these categories were completely irrelevant, their lack of association with other categories suggests that they are “dead-end,” as in there are no pathways from which these categories can be altered by human factors interventions directed at other latent condition categories within the system. By comparing which categories are dead-end and which categories are associated with a wider range of conditions, safety practitioners can improve the efficiency of human factors interventions by acting through categories which are linked with a greater number of safety outcomes.

To illustrate, present results for the low experience group found six associations between active failures (L1) and higher-level latent conditions (across levels 2–4) (Table 4; Figure 2). This would suggest that level 1 active failures for the low experience group can be resolved across six categories of latent conditions. Hence, simple, functionalist interventions on rectifying frontline goals are likely to be suitable—there are six categories across the various levels within the organization which can be modified to result in desired changes. On the other hand, for the high experience group, “Supervisory Violations” at level 3 was the only category that was associated with level 1 active failures (Table 5). This is far fewer than the six categories in the low experience group. Human factors interventions focusing solely on the rectification of supervisory violations at level 3 are unlikely to be successful as they will be constrained by the concerns, influences, and actions of factors at other levels, such as organizational (L4) climate and processes. In this case, anthropological interventions may be more ideal as outside-in changes can act through the meta-categories of concerns, influences, and actions to change the interactive interfaces across all levels.

### 5.5. Practical applications

At present, SMS functions within airlines and aviation operators need to solve several challenges. The first challenge is to establish a method to make the HFACS categories more relevant across different target groups. This will help to ensure that the threats and precursors included in the SMS are relevant to the professional context or environment. The present research has demonstrated that the classification of threats can be influenced by professional experience. In practice, this supports the integration of demographic variables such as experience into the SMS. For example, if the target users are pilots with high experience, then a focus on managing high-level conditions may be more relevant when coming up with safety objectives in the initial stages of the SMS process.

The second challenge in SMS implementation is in establishing what type of interventions are relevant and efficient. This is important in most real-world organizational settings as resource constraints will typically limit the number and the variety of interventions that can be carried out. To aid in selecting interventions that are more likely to be successful, the present results suggest placing a higher level of attention toward categories with many paths of association with other categories. In practice, the consideration of how each category interacts with other categories through the threads of *concerns, influences*, and *actions* are recommended to ensure that a variety of mitigation strategies within the HFIX framework are covered.

Human factors interventions which can simultaneously alleviate concerns, influences, and actions across multiple system levels are likely to satisfy more of the HFIX criteria. In contrast, interventions directed at causal condition categories which are either unassociated with other categories, or are ringfenced by external concerns, influences, and actions may be less effective. To illustrate, in the present study the low experience group associated “Violations” with “Planned Inappropriate Operations” and “Adverse Mental States.” Interventions focusing solely on *actions* (violations) are unlikely to be successful unless supervisory *influences* (planning of operations) and worker *concerns* (mental states) are addressed. Applied to the HFIX framework, this would imply that human/crew mitigation strategies to fix violations may be futile unless both task/mission and operational/physical environment changes are established.

### 5.6. Limitations and future research

A limitation of the present study is that the demographic variables were not fully controlled. Although TFH is a proven measure of pilot experience and was valuable in showcasing the effects of power distance in the present research, it was not possible to completely ensure that other professional experience factors did not confound the results. For example, some high TFH pilots may elect to work in a lower ranking position, and this may confound the validity of TFH as a proxy for rank and position. Moreover, as the data was collected during the COVID-19 pandemic during which many pilots were furloughed or demoted to junior ranks (39), the present data on rank and qualification may not be truly representative and any future research will require further data collection. Similarly, as job demands and workload have changed throughout the pandemic (40), the influence of flight experience levels may have also been affected. In the future, it will be interesting to conduct multi-level analysis to segregate the effects of the various demographic variables.

Another limitation of the present study is the question of whether high and low experienced pilots were different enough to be considered as two distinct demographic groups. Regardless of how previous research justified the use of TFH as a variable by associating experience with occupational immersion and expertise, an opportunity for future research will be to expand the comparisons to other professional groups. For example, values and attitudes of aviation industry professionals were known to differ between pilots, cabin crew, ground staff, and airline managers (5). As the limitation of using pilots in the present study arose because this paper was part of a project evaluating SMS in airline flight operations departments, a possibility for the future will be to expand data collection and analysis to other departments within the organization.

## 6. Conclusion

The present research compared HFACS classification between pilots with high and low experience, as measured by total flight hours (TFH). Significant differences in the use of HFACS categories, and in paths of association between pairs of categories were found between the high and low experience groups. The low experience group cast a wider net, with active failures at the frontline (L1) associated with a greater number of categories at levels 2–4. The low experience group were also comparatively more affected by stress and uncertainty, with a strong association between “Adverse Mental States” and active failures. On the other hand, high TFH pilots were more targeted in identifying patterns of associations between unsafe acts and their causal conditions. The high experience group also had a greater tendency to classify causal factors as originating from higher-level supervisory or organizational conditions. Notably, across both high and low experience groups, most of the significant associations were between categories which were not at immediately adjacent system levels, suggesting that cross-level interactions through the threads of concerns, influences, and actions can influence the perceived accountabilities and thereby affect hazard and risk evaluation in SMS. As these differences will consequently impact the selection of safety interventions, the present results make a strong case for the inclusion of the target users’ demographic variables in the earlier, investigative stages of the SMS. Human factors interventions focusing on functionalist changes to tangible behaviors are likely to be more suitable for the low experience group, whereas anthropological changes to cultural meanings and patterns which can originate from wider factors outside of the organization will be more suitable for the high experience group. Findings of the present research will contribute to organizations in aviation and other high reliability industries by encouraging safety managers to consider demographic experience effects on the classification and association of human factors conditions. The integration of human factors classification with open-system concepts of safety culture and safety practices will also provide SMS practitioners with the innovative ability to select more effective intervention and safety management approaches.

## Data availability statement

The datasets presented in this study can be found in online repositories. The names of the repository/repositories and accession number(s) can be found in the article/Supplementary material.

## Ethics statement

The studies involving human participants were reviewed and approved by the Cranfield University Research Ethics Committee (CURES 12290/2020). The patients/participants provided their written informed consent to participate in this study.

## Author contributions

WC worked with W-CL to develop the method for data collection, data analysis, and writing the manuscript. WC contributed to literature review and data collection from airlines and was working in aviation domain. W-CL facilitated the discussion section with WC and drafted the conclusion together, and communicated with journal administrator for the submission processes. All authors contributed to the article and approved the submitted version.

## Funding

This study was supported by Cranfield University library's open access agreement.

## Conflict of interest

The authors declare that the research was conducted in the absence of any commercial or financial relationships that could be construed as a potential conflict of interest.

## Publisher’s note

All claims expressed in this article are solely those of the authors and do not necessarily represent those of their affiliated organizations, or those of the publisher, the editors and the reviewers. Any product that may be evaluated in this article, or claim that may be made by its manufacturer, is not guaranteed or endorsed by the publisher.

## Supplementary material

The Supplementary material for this article can be found online at: https://www.frontiersin.org/articles/10.3389/fpubh.2023.1144921/full#supplementary-material

## Footnotes

^1^ eu.qualtrics.com

## References

[1] ICAO. Safety Management Manual. [Document 9859]. International Civil Aviation Organization. Montreal, Canada (2018)

[2] Dekker SWA. The re-invention of human error. Hum Fact Aerosp Saf. (2001) 1:247–65.

[3] Aydin M Uğurlu Ö Boran M. Assessment of human error contribution to maritime pilot transfer operation under HFACS-PV and SLIM approach. Ocean Eng. (2022) 266:112830. doi: 10.1016/j.oceaneng.2022.112830

[4] Baumann MR Sniezek J Buerkle CA. Self-evaluation, stress and performance: a model of decision making under acute stress In: Salas E Klein G, editors. Linking expertise and naturalistic decision making. 1st ed: Psychology Press (2001)

[5] Chan WT-K Li W-C. Investigating professional values among pilots, cabin crew, ground staff, and managers to develop aviation safety management systems. Int J Ind Ergon. (2022) 92:103370. doi: 10.1016/j.ergon.2022.103370

[6] Klein G. Naturalistic decision making. Hum Fact. (2008) 50:456–60. doi: 10.1518/001872008X288385 18689053

[7] ETSC. Transport safety performance indicators. Etterbeek, Belgium: European Transport Safety Council (2001) Available at: http://archive.etsc.eu/documents/perfindic.pdf

[8] Wiegmann DA Shappell SA. A human error approach to aviation accident analysis. Oxon, England: Routledge (2003).

[9] Morley FJJ Harris D. Ripples in a pond: an open system model of the evolution of safety culture. Int J Occup Saf Ergon. (2006) 12:3–15. doi: 10.1080/10803548.2006.11076666, PMID: 16553996

[10] Shappell S Wiegmann D. A methodology for assessing safety programs targeting human error in aviation. Int J Aviat Psychol. (2009) 19:252–69. doi: 10.1080/10508410902983904

[11] Simard M Marchand A. Workgroups’ propensity to comply with safety rules: the influence of micro-macro organisational factors. Ergonomics. (1997) 40:172–88. doi: 10.1080/001401397188288

[12] Helmreich RL Merritt AC. Culture at work in aviation and medicine In: . Culture at work in aviation and medicine. 1st ed: London, England: Routledge (2019) 27–52.

[13] Li W.-C. Harris D. Li L.-W. Hsu Y.-L. Wang T. (2010). The investigation of accidents related to aeronautical decision-making in flight operations. Proceedings of the 41st Annual International Seminar: Investigating ASIA in Mind–Accurate, Speedy, Independent, and Authentic, (Sterling, Virginia, USA: International Society of Air Safety Investigators). 82–88.

[14] McDonald N Ryan F. Constraints on the development of safety culture: a preliminary analysis. Ir J Psychol. (1992) 13:273–81. doi: 10.1080/03033910.1992.10557886

[15] Harris MR Fein EC Machin MA. A systematic review of multilevel influenced risk-taking in helicopter and small airplane Normal operations. Front Public Health. (2022) 10:823276. doi: 10.3389/fpubh.2022.823276 35646790 PMC9133595

[16] Burian BK Orasanu J Hitt J. Weather-related decision errors: differences across flight types. Proc Hum Fact Ergonom Soc Annu Meet. (2000) 44:22–5. doi: 10.1177/154193120004400107

[17] Li G Baker SP Grabowski JG Rebok GW. Factors associated with pilot error in aviation crashes. Aviat Space Environ Med. (2001) 72:52–8. PMID: 11194994

[18] Bazargan M Guzhva VS. Impact of gender, age and experience of pilots on general aviation accidents. Accid Anal Prev. (2011) 43:962–70. doi: 10.1016/j.aap.2010.11.023, PMID: 21376889

[19] Rebok GW Qiang Y Baker SP McCarthy ML Li G. Age, flight experience, and violation risk in mature commuter and air taxi pilots. Int J Aviat Psychol. (2005) 15:363–74. doi: 10.1207/s15327108ijap1504_4

[20] Goh J Wiegmann DA. Relating flight experience and pilots’ perceptions of decision-making skill. Proc Hum Fact Ergonom Soc Annu Meet. (2002) 46:81–5. doi: 10.1177/154193120204600117

[21] Yang JH Kennedy Q Sullivan J Fricker RD. Pilot performance: assessing how scan patterns and navigational assessments vary by flight expertise. Aviat Space Environ Med. (2013) 84:116–24. doi: 10.3357/ASEM.3372.2013 23447849

[22] Antonsen S. (2012). Safety culture: theory, method and improvement. London, England: CRC Press.

[23] Bienefeld N Grote G. Silence that may kill. Aviat Psychol Appl Hum Fact. (2012) 2:1–10. doi: 10.1027/2192-0923/a000021

[24] Todd MA Thomas MJW. Flight hours and flight crew performance in commercial aviation. Aviat Spac Environ Med. (2012) 83:776–82. doi: 10.3357/asem.3271.2012, PMID: 22872992

[25] Salas E Burke CS Fowlkes JE Wilson KA. Challenges and approaches to understanding leadership efficacy in multi-cultural teams In: Kaplan M, editor. Cultural ergonomics advances in human performance and cognitive engineering research, vol. 4: Emerald Group (2004). 341–84.

[26] Chen J.-C. Lin S.-C. Li W.-C. (2014). Utilization of Human Factors Intervention Matrix (HFIX) to develop aviation safety management strategy. Proceedings of the 18th World Multi-Conference Systemics, Cybernetics and Informatics, 2014, 50–55. Orlando, FL.

[27] Hudson P. (1999). Safety culture-theory and practice. The Human Factors in System Reliability-Is Human Performance Predictable? Siena.

[28] Naevestad TO. Mapping research on culture and safety in high-risk organizations: arguments for a sociotechnical understanding of safety culture. J Contingencies Crisis Manag. (2009) 17:126–36. doi: 10.1111/j.1468-5973.2009.00573.x

[29] Tharaldsen J Haukelid K. Culture and behavioural perspectives on safety – towards a balanced approach. J Risk Res. (2009) 12:375–88. doi: 10.1080/13669870902757252

[30] Tear MJ Reader TW Shorrock S Kirwan B. Safety culture and power: interactions between perceptions of safety culture, organisational hierarchy, and national culture. Saf Sci. (2020) 121:550–61. doi: 10.1016/j.ssci.2018.10.014

[31] Guo Z Rau P-LP Heimgärtner R. The “onion model of human factors”: a theoretical framework for cross-cultural design In: Atthia Rauterberg M, editor. Culture and computing. HCII 2022, Lecture Notes in Computer Science, vol. 13324. Cham: Springer (2022). 20–33.

[32] Edwards JRD Davey J Armstrong K. Returning to the roots of culture: a review and re-conceptualisation of safety culture. Saf Sci. (2013) 55:70–80. doi: 10.1016/j.ssci.2013.01.004

[33] BFU. (2004). Investigation report AX001-1-2/02 (accident 1 July 2002 near Ueberlingen/Lake of Constance/Germany). Germany:Bundesstelle für Flugunfalluntersuchung [Federal Bureau of Aircraft Accidents Investigation].

[34] Brooker P. The Überlingen accident: macro-level safety lessons. Saf Sci. (2008) 46:1483–508. doi: 10.1016/j.ssci.2007.10.001

[35] Li W-C Young H-T Wang T Harris D. International cooperation and challenges: understanding cross-cultural issues. ISASI Forum. (2007) 40:16–21.

[36] Bennett S. The 1st July 2002 mid-air collision over Öberlingen, Germany: a holistic analysis. Risk Manage. (2004) 6:31–49. doi: 10.1057/palgrave.rm.8240171

[37] Masys AJ. A systemic perspective of situation awareness: an analysis of the 2002 mid-air collision over Überlingen, Germany. Disast Prevent Manag. (2005) 14:548–57. doi: 10.1108/09653560510618375

[38] Nunes A Laursen T. Identifying the factors that contributed to the Ueberlingen midair collision. Proc Hum Fact Ergonom Soc Annu Meet. (2004) 48:195–8. doi: 10.1177/154193120404800142

[39] Mizzi A Lohmann G Carim G Jr. Clipped wings: the impact of the Covid-19 pandemic on airline pilots and influence on safety climate In: Harris D Li W-C, editors. Engineering psychology and cognitive ergonomics. HCII 2022, Lecture Notes in Computer Science, vol. 13307. Cham: Springer (2022)

[40] Vuorio A Bor R. Black swan pandemic and the risk of pilot suicide. Front Public Health. (2020) 8:573006. doi: 10.3389/fpubh.2020.573006 33224917 PMC7671189

